# Cochlear Delay and Medial Olivocochlear Functioning in Children with Suspected Auditory Processing Disorder

**DOI:** 10.1371/journal.pone.0136906

**Published:** 2015-08-28

**Authors:** Sriram Boothalingam, Chris Allan, Prudence Allen, David Purcell

**Affiliations:** 1 National Center for Audiology, Western University, London, ON, Canada; 2 School of Communication Sciences and Disorders, Western University, London, ON, Canada; Sun Yat-sen University, CHINA

## Abstract

Behavioral manifestations of processing deficits associated with auditory processing disorder (APD) have been well documented. However, little is known about their anatomical underpinnings, especially cochlear processing. Cochlear delays, a proxy for cochlear tuning, measured using stimulus frequency otoacoustic emission (SFOAE) group delay, and the influence of the medial olivocochlear (MOC) system activation at the auditory periphery was studied in 23 children suspected with APD (sAPD) and 22 typically developing (TD) children. Results suggest that children suspected with APD have longer SFOAE group delays (possibly due to sharper cochlear tuning) and reduced MOC function compared to TD children. Other differences between the groups include correlation between MOC function and SFOAE delay in quiet in the TD group, and lack thereof in the sAPD group. MOC-mediated changes in SFOAE delay were in opposite directions between groups: increase in delay in TD vs. reduction in delay in the sAPD group. Longer SFOAE group delays in the sAPD group may lead to longer cochlear filter ringing, and potential increase in forward masking. These results indicate differences in cochlear and MOC function between sAPD and TD groups. Further studies are warranted to explore the possibility of cochlea as a potential site for processing deficits in APD.

## Introduction

“Auditory Processing Disorder (APD) refers to difficulties in the perceptual processing of auditory information in the central nervous system and the neurobiological activity that underlies the processing” [1]. Difficulty hearing speech in noise despite normal hearing thresholds is the hallmark of APD [2]. APD is a heterogeneous disorder involving a breakdown of various aspects of auditory processing, resulting in complaints and symptoms that vary remarkably across the population [3]. Problems at various levels of the auditory neural system in both the afferent (bottom-up) pathway and in the higher level processing that fine-tunes afferent pathways via efferent connections (top-down) have been reported in APD [1]. For example, studies have shown atypical signal encoding at the brainstem (e.g., Hornickel et al. [4] and Allen and Allan [5]), midbrain (e.g., Purdy et al. [6]), and the cortex (e.g., Abrams et al. [7]) in children with listening problems. However, cochlear functioning remains relatively unexamined in these children.

Peripheral auditory mechanisms are not typically included in the diagnosis of APD, rather, they are only screened for the presence of an overt hearing loss. However, the cochlea performs substantial signal processing that is crucial for speech perception. Cochlear tuning is directly related to frequency selectivity and temporal processing ability [8], and therefore is important for good speech discrimination, especially in noise [9]. Impaired cochlear tuning causes increase in masking and reduces suppression, which can in turn reduce contrasts between speech sounds and affect speech perception [10]. Although cochlear tuning impairments are typically seen in individuals with cochlear hearing loss, Badri et al. [11] reported broader tuning in normal hearing adults who complained of poor speech discrimination in noise. Patterson et al. [12] previously showed that cochlear tuning deteriorated with age, despite clinically normal hearing sensitivity. Findings from these studies suggest that conventional audiograms and speech tests in quiet do not capture subtle deficits in cochlear functioning that can impact speech perception in noise, which is typical of APD. Although reduced tuning is certainly linked to impaired speech perception, it has also been shown that sharper tuning impacts temporal aspects of speech perception [13]. As such, there appears to be a range of optimal cochlear filter widths for auditory processing. To explore this range in tuning and better understand cochlear processing in children with listening problems, an objective physiological measure of cochlear tuning, stimulus frequency otoacoustic emission (SFOAE) group delay [14, 15], was used in the present study.

Cochlear processing does not happen in isolation, it is influenced by corticofugal connections (top-down) via the medial olivocochlear system (MOC) (e.g., Dragicevic et al. [16]). MOC axons innervate outer hair-cells (OHCs) directly, and inhibit OHC electromotility via cholinergic synapses. This action causes a reduction in cochlear amplification, and can be quantified by measuring changes in otoacoustic emission (OAE) level in the earcanal (review: Guinan [17]). OAEs are byproducts of the cochlear amplification process and thus are thought to reflect cochlear activity at their generation site. Reduction in cochlear amplification also alters cochlear filter tuning [18]. Such subtle changes have been hypothesized to affect pitch perception and localization abilities [18].

MOC activity is of particular interest in the APD population because it is thought to aid in unmasking signals from noise [17]. Some studies have reported that the MOC unmasking function is reduced in individuals with listening difficulties [19, 20], while others do not [21, 22]. Thus, there is no clear consensus on MOC function in individuals with listening difficulties possibly associated with APD. However, previous MOC assays have only investigated gross changes in OAE level. MOC mediated change in other attributes such as cochlear filter delay remain unrecognized. Subtle discrepancies in cochlear processing due to MOC activation may be reflected better in a filter-delay metric, and thus may augment findings of OAE level changes. Therefore, we chose to measure MOC inhibition of SFOAE level and delay to characterize MOC functioning in children suspected with APD in the present study. SFOAEs are generated from a narrow site on the basilar membrane at low stimulus levels [23]. Frequency specificity of the SFOAEs aid in understanding the changes that the MOC produces in the cochlea.

## Methods

### Participants

Sixty three children in the age range 7-17 years took part in the study. Thirty eight children were referred to our in-house Audiology clinic with listening problems (suspected APD group: sAPD, mean age: 9.13 years, standard deviation [SD]: 2.39 years, 8 females) and twenty five were typically developing children with no complaints in listening (TD group, mean age: 11.59 years, SD: 2.46 years, 13 females). All children had normal middle ear function as determined by clinical tympanometry (GSI-TympStar, Grason-Stadler Inc., MN) and hearing thresholds of 20 dB HL or better at octave intervals between 0.25 and 8 kHz measured using a clinical audiometer (GSI-61, Grason-Stadler Inc., MN). All children had contralateral acoustic reflex thresholds >70 dB HL for steady state broadband noise (BBN). Children also underwent a screening distortion product OAE (DPOAE) measurement (Integrity v-500, Vivosonic Inc., ON) to confirm the presence of OAEs.

Children in the sAPD group underwent a test battery similar to that used by Allen and Allan [5] that included three standard clinical tests: the Staggered Spondaic Word Test (SSW; Katz [24]), Words in Ipsilateral Competition (WIC; Ivey [25]) and Pitch Pattern Sequence test (PPS; Pinheiro [26]), and two psychoacoustic tests that use adaptive procedures developed in-house for use with children: Gap Detection (GD), and Difference Limen for Frequency (DLF) as well as the click evoked auditory brainstem response (ABR). Tests were administered in accordance with their respective manuals and were interpreted according to published age-specific normative data. Of the 38 children in the sAPD group, 27 were diagnosed as having APD based on American Speech-Language Hearing Association (ASHA) guidelines [27], i.e., scored 2 SDs below the normative expectation in at least two tests. Seven children failed in one test, and four children passed all tests. Of the 11 children who passed all or all-but-one behavioral measures, all had atypical ABR in the form of prolonged peak latencies, prolonged inter-peak latencies, or abnormal wave I-V amplitude ratio. Abnormalities in ABR have been reported in children suspected with APD. A recent study [5] showed that behavioral tests alone may not be adequate in diagnosis of APD, which supports recommendations by professional bodies (e.g., American Academy of Audioligy (AAA) [1]). Allen and Allan [5] found several children who passed these behavioral tests had abnormal neural encoding of sound measured using ABR and/or absent/elevated acoustic reflex thresholds. Therefore, children who passed the behavioral test battery but who had abnormal ABR were also included in the study group (sAPD) along with children diagnosed as APD using the behavioral test battery.

Participants sat in a comfortable chair in a double-walled sound attenuated booth (Eckel Industries, ON) and watched a silent closed captioned movie. They were encouraged to relax, and swallow as few times as comfortable. SFOAEs were recorded from only one ear per participant. The ear being tested was chosen based on (larger) DPOAE amplitude obtained during the screening process. Study methods were approved by the Health Sciences Research Ethics Board of Western University, Canada (approval #17731E). The nature of the study was explained prior to obtaining written informed assent from every participant, and a written informed consent from participants’ parent/caregiver. Participants were compensated for their time with gift cards towards books or school supplies.

### SFOAE measurement

#### Stimulus and instrumentation

All signals were generated digitally in Matlab (Mathworks, MA) at a sampling rate of 32 kHz and at a bit depth of 24. SFOAEs were evoked by 2.048 s probe tones presented at 40 dB SPL, with probe frequencies (*f_P_*) ranging from 0.928 to 1.248 kHz at 16 Hz intervals. Measurements focused on the 1 kHz region because this is where MOC effects are reported to be the strongest [28]. Suppressor tones (*f_S_*) corresponding to each *f_P_* (where, *f_S_* = *f_P_* + 16 Hz) of 2.048 s with linear rise/fall ramps of 50 ms duration and 60 dB SPL in level were used according to the suppression method [29] to extract SFOAEs using discrete Fourier transforms (explained below). Frequencies of all tones were adjusted to have an integer number of cycles in the analysis window. The MOC elicitor was uniform random unfiltered broadband noise (BBN), which had a flat spectrum from 0 to 10 kHz. BBN was presented at 60 dB SPL, which was at least 10 dB below the ART of all participants.

Signals were presented through a digital-to-analog converter (6289 m-series; National Instruments, TX) at a sampling rate of 32 kHz to three individual programmable attenuators (PA5; Tucker-Davis Technologies, FL) that controlled the output signal levels of the probes, suppressors and elicitors. Signals were then power amplified (SA1; Tucker-Davis Technologies, FL) and fed to ER2 transducers (Etymotic Research, IL) connected to an ER-10B+ otoacoustic emission probe system (Etymotic Research, IL) that delivered the signals in the ear-canal.

The ear-canal pressure was recorded using the ER-10B+ probe system with a pre-amplifier gain of +40 dB. The recorded signal was bandpass filtered (Frequency Devices Inc., IL) between 0.4 and 10 kHz and a gain of 20 dB was applied. The filtered signal was then digitized by an analog-to-digital converter (6289 m-series; National Instruments, TX) which applied another 6 dB of gain prior to conversion. Stimulus delivery and response acquisition were controlled using custom programs developed in LabView (National Instruments, TX). All stimuli were calibrated using a Type-2250 sound level meter (Brüel and Kjær, Denmark), and an ear simulator Type-4157 (IEC 711; Brüel and Kjær, Denmark).

#### SFOAE recording

**Fig 1:**
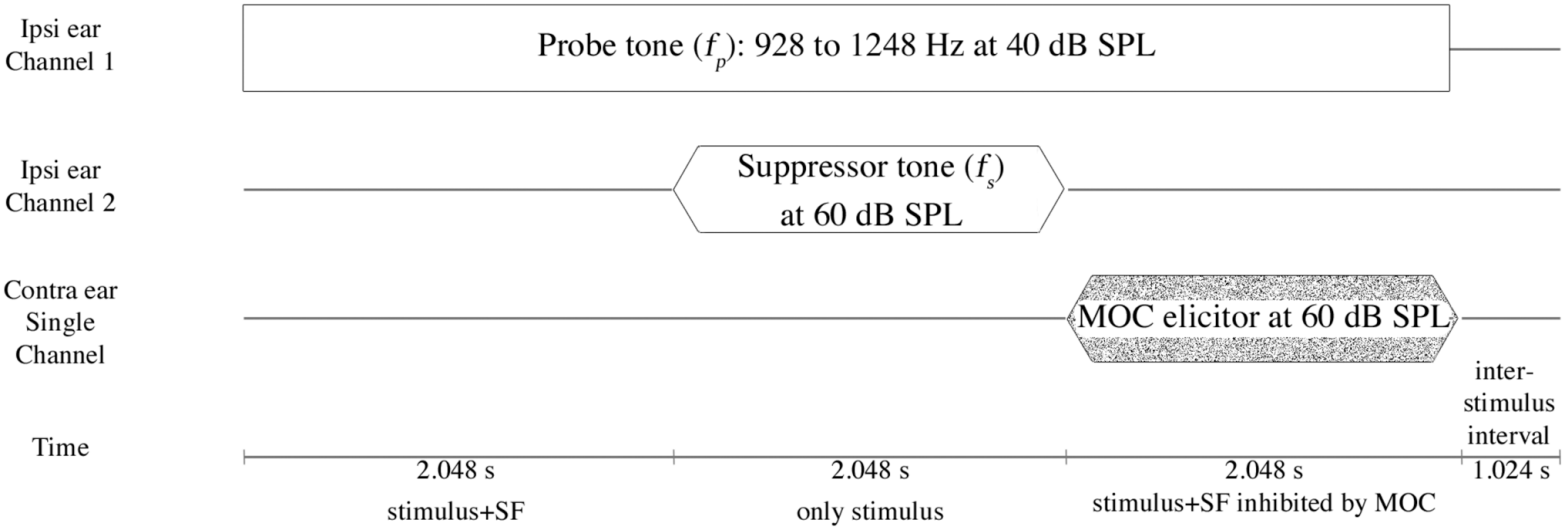
Schematic representation of SFOAE paradigm. Schematic representation and temporal sequence of events for SFOAE recorded with and without MOC elicitors. Channels in the left most column indicate separate physical transducers. Duration of each sweep-block are mentioned in the last row. *f_P_* was presented continuously while the suppressor *f_S_* was turned on in the second sweep-block. MOC elicitor was turned on in the third sweep-block to obtain MOC inhibited *f_P_*. Note that size of each element in the figure is made disproportionate to their duration to show shorter events clearly.

To describe each stimulus, we will use the terms ‘epoch’, ‘sweep-block’ and ‘sweep’ (see Fig 1). An epoch was 1.024 s in duration and sweep-blocks were made of two consecutive epochs of the same stimulus. Multiple sweep-blocks were concatenated to create a sweep of 7.168 s in duration. One complete sweep had three sweep-blocks: 1. *f_P_* in isolation, 2. *f_P_* with *f_S_*, and 3. *f_P_* with elicitor. A 1.024 s inter-sweep interval was used between sweeps to ensure that the MOC reverted to its baseline activity [30]. An in-the-ear calibration of the tones was carried out before every measurement to produce the desired (*f_P_*) SPL in the ear-canal. Each frequency *f_P_* was repeated for at least five sweeps to obtain reliable SFOAEs. Additional sweeps were recorded: if noisy epochs (if the epoch root-mean-square [RMS] amplitude exceeded 0 dB SPL in a 0.5 to 0.9 kHz band) were detected, for clipped epochs, or if the SNR (SFOAE vs. noise floor) was lower than 10 dB. A maximum of 5 additional sweeps could be collected in a noisy participant.

#### SFOAE extraction

Total ear canal pressure (*P_ECtot_*) in the first sweep-block contained both the probe stimulus, *f_P_*, and the SFOAE as a composite mixture. The intracochlear suppressor, *f_S_*, was then presented in the second sweep-block, in addition to the *f_P_*, to maximally suppress the generated SFOAE (*P_ECms_*) [29]. A vector subtraction of the average ear canal pressure (at *f_P_*) in sweep-blocks 1 and 2 yielded the baseline SFOAE (*P_SF_*), i.e., SFOAE in elicitor-off condition:

$$\begin{array}{c} P_{SF}\left(\text{baseline}\right)=P_{ECtot}\left(\text{sweep-block}\;1\right)-P_{ECms}\left(\text{sweep-block}\;2\right) \end{array}$$ (1)

Similarly, vector subtraction of the sweep-blocks 2 and 3 yielded SFOAE with MOC inhibition, i.e., elicitor-on condition:

$$\begin{array}{c} P_{SF}\left(\text{MOC-inhibited}\right)=P_{ECtot}\left(\text{sweep-block}\;3\right)-P_{ECms}\left(\text{sweep-block}\;2\right) \end{array}$$ (2)

MOC strength was defined as the difference in SFOAE level between baseline (elicitor-off) and elicitor-on conditions. The difference is calculated in a linear scale (Pascal) and converted to (1) a dB change metric, and (2) as a percentage change in elicitor-on condition from baseline. Where, percent change is the difference in SFOAE level between the two conditions normalized by the baseline SFOAE level (all in Pascals) and multiplied by 100. SFOAE stimulus levels were chosen based on previous studies [31, 32] to obtain good signal-to-noise ratio (SNR) while avoiding the SFOAE stimulus from evoking any ipsilateral MOC activity. The first and last 128 ms of every response were discarded to avoid transients that may have occurred due to stimulus onset/offset. Following online SFOAE extraction, all epochs were again evaluated offline using a discrete Fourier transform to obtain noise metrics in a 20 Hz band just below *f_P_*. Epochs with noise metrics that exceeded the mean plus two SDs were not included in the average response sweep. Also, SFOAE level enhancements due to MOC activation were not considered. Such enhancements may not be true MOC effects, they may occur due to change in phase relationship between the reverse traveling evoked-OAE and forward traveling OAE reflected from the middle-ear boundary [33]. Spontaneous OAEs (SOAEs) were recorded to allow rejection of SFOAEs within 50 Hz of an SOAE to avoid phase related complexities.

#### An SFOAE-based measure of cochlear delay

A measure of cochlear delay, *τ*, was obtained from the negative slope of the SFOAE phase gradient (rate of change of SFOAE phase as a function of frequency). This has been shown to reflect the round-trip propagation time: the time taken for *f_P_* to reach its characteristic frequency (CF) place on the basilar membrane, and for the generated emission to return to the ear-canal [15]. The traveling wave build-up near the CF however accounts for the bulk of time during this round-trip propagation, while the ear-canal, middle-ear and basilar membrane transmission times are negligible [34]. For these reasons, *τ*, which is roughly half of the total time taken, serves as an indirect measure of cochlear delay. Based on the filter theory [35] and coherent reflection filtering theory [14], SFOAE delay is an analogue of the cochlear filter delay. Sharper filters ring longer and produce delayed outputs compared to broader filters. Therefore, if cochlear tuning is sharp, one would expect longer SFOAE delay, as SFOAEs are generated by coherent reflection of random irregularities close to the peak of the traveling wave [14].

The phase slope of SFOAEs and the bandwidth (BW) of the cochlear filter at a given frequency are thus inversely related, with steeper phase slopes corresponding to sharper cochlear tuning [15]. This method of calculation of cochlear delay has been implemented in several animal models (e.g., Bergevin et al. [36]) and humans (e.g., Bhagat and Kilgore [37], Boothalingam and Lineton [38], and Guinan et al. [32]). The *τ* in the present study was measured in a similar fashion to Boothalingam and Lineton [38]. Only frequency regions that had SNRs better than 10 dB were considered for obtaining the group delay. This was achieved by the first author manually picking only the best SNR region (>10 dB); a linear regression line was then fit in that band to obtain *τ*. At least 5 consecutive frequency points were required to obtain a line fit.

It should be noted that for a given sharpness of a filter, SFOAE delays generated within this region will differ based on the distribution of reflecting elements [39]. Distribution of such reflecting elements are thought to be random along the basilar membrane, leading to inherent variability in *τ* across individuals. There is also some debate around the validity of deriving tuning from OAE delays, for example, due to uncertainties in SFOAE delays measured at low frequencies [40]. Nonetheless, the current study is comparative, involving one method to compare tuning between sAPD and TD groups. Therefore statistically significant differences in *τ* between the groups could indeed mean differences in their cochlear function.

### Test for Middle-Ear Muscle Reflex (MEMR)

Activation of MEMR also causes reduction in SFOAE level, similar to MOC, albeit at slightly higher stimulus levels. It is prudent to avoid activating this reflex in order to establish a causal relationship between MOC activation and corresponding reduction in SFOAE level in the contralateral ear. In addition to confirming that ARTs for all children were >70 dB HL using a clinical immitance meter, additional analyses were performed to rule out MEMR. This additional check was performed in light of several recent studies showing that MEMR can be activated at levels much lower than what is typically obtained with a clinical immitance meter [32]. The present test is based on the hypothesis that a significant MEMR would consistently increase probe-tip stimulus levels. This is because, MEMR activation will stiffen the ossicular chain and retract the tympanic membrane, resulting in increased reflection of stimulus energy back to the ear-canal. A cut-off value of 1.4% (0.12 dB) increase in stimulus level during elicitor-on condition compared to no-elicitor condition has been suggested as an indication of MEMR activation [41, 42].

**Fig 2:**
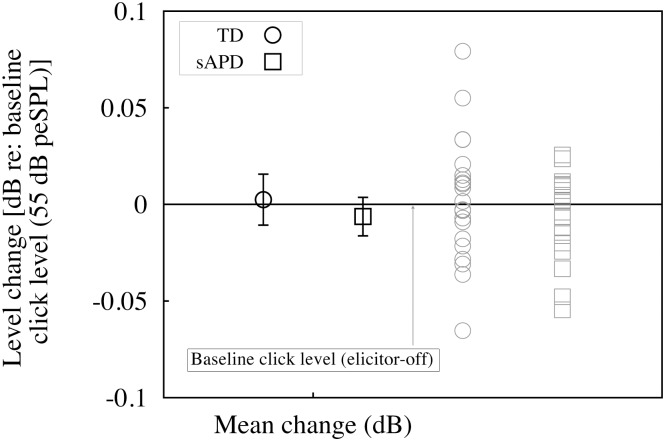
Results of MEMR test. Means and their corresponding individual data for the change in stimulus level with reference to baseline no-elicitor condition (dB) is plotted (Y-axis). Black straight line at 0 dB represents normalized baseline stimulus level (in no-elicitor condition). Black symbols are group means with their corresponding 95% confidence intervals represented by error bars. Grey symbols are individual means of RMS amplitude near the stimulus trough. Circles represent TD and boxes represent sAPD groups, respectively.

To test for such changes in level, 55 dB peSPL clicks presented at 41.67 Hz were used as probe stimuli. Clicks are more potent elicitors of the MEMR [32], therefore if MEMR was not evoked for clicks, it can be assumed that MEMR will not be evoked by the SFOAE evoking puretones. Clicks were recorded in the ear-canal in two conditions, one without any contralateral elicitor and one with the same contralateral elicitor used in the study to elicit MOC reflex. RMS levels of the ear-canal recorded clicks were obtained for every participant in a time-window (125 *μ*s long) near the first trough of the click waveform for elicitor-on/off conditions. The first trough is the first, and the largest, deviation in sound pressure that was measured in the earcanal that occurs due to ringing of the click stimulus. The trough lasts roughly for 125 *μ*s, and this response was windowed for observing changes that may have occurred due to MEMR. As seen in Fig 2, changes in the presence of MOC elicitors were on average −0.0019 dB ±0.008 (re: baseline no-elicitor). Observed stimulus level deviations occur in both directions, i.e., increase and decrease in level with the largest change being <0.08 dB. The observed changes are small compared to level changes that would be expected if the MEMR was activated, i.e., >1.4% (0.12 dB) [41]. These changes probably arise due to random fluctuations in background noise. Note that five children (1 from TD, and 4 from sAPD group) did not undergo this secondary MEMR test due to time constraints. Therefore, in these children, their ART thresholds alone were used for the evaluation of MEMR activation.

It should be noted that energy reflectance due to MEMR can increase or decrease beyond ∼ 1.2 kHz [43]. However, Boothalingam et al. [44] used a similar test for 1 and 2 kHz tonebursts (presented at 55 dB peSPL) and reported similar stimulus changes across participants for both frequencies. Considering the bandwidth of the clicks in the current test (∼ 2.5 kHz [3 dB down rule], and ∼ 6 kHz [10 dB down rule]), most click energy can be expected to be concentrated within this (0–2.5 kHz) frequency range. Therefore, difference in reflectance change with frequency may not have had a substantial effect on the stimulus-level-change values reported for the MEMR test. Therefore, all frequencies (0.5 to 6 kHz) were considered in the current method in order to conserve SNR.

### Data inclusion criteria

For data to be considered for statistical analyses the following criteria had to be met: (1) <10% epoch rejection, (2) minimum SNR of 10 dB, and (3) no MEMR activation. Based on the inclusion criteria, 18 participants (15 from the sAPD group and 3 from the TD group) were rejected from the study mainly due to excessive participant-related artifacts leading to poor SNR. This led to inclusion of 22 participants in the TD and 23 in the sAPD group. Noise floor analysis was performed with the rejected data to explore the reasons for rejection in detail.

## Results

Prior to any data analysis, group difference in age was probed for included participants. Independent sample *t*-test indicated a significant difference (Mean difference [MD] = 1.98, 95% confidence interval (CI_95%_) = ±1.59 years, *t*[43] = 2.52, *p* = 0.02) in age between TD and sAPD groups (TD>sAPD). However, univariate analysis of variance results show that age is not a significant predictor of both SFOAE level (*F*[1, 43] = 1.16, *p* = 0.29) and *τ* (*F*[1, 43] = 1.29, *p* = 0.26). In addition, previous studies have indicated that both cochlear and MOC function are mature at full-term birth [41, 45]. Therefore, the observed difference in age between the two groups should not have a bearing on any group differences reported below.

There were also no significant differences in puretone thresholds between TD and sAPD groups (MD = 1.94, CI_95%_ = ±2.66 dB HL (TD>sAPD) *t*[39] = 1.47, *p* = 0.149). Note that puretone averages of four children in the TD group were unavailable, as they were only screened for thresholds <20 dB HL.

### MOC-mediated change in SFOAE level

**Fig 3:**
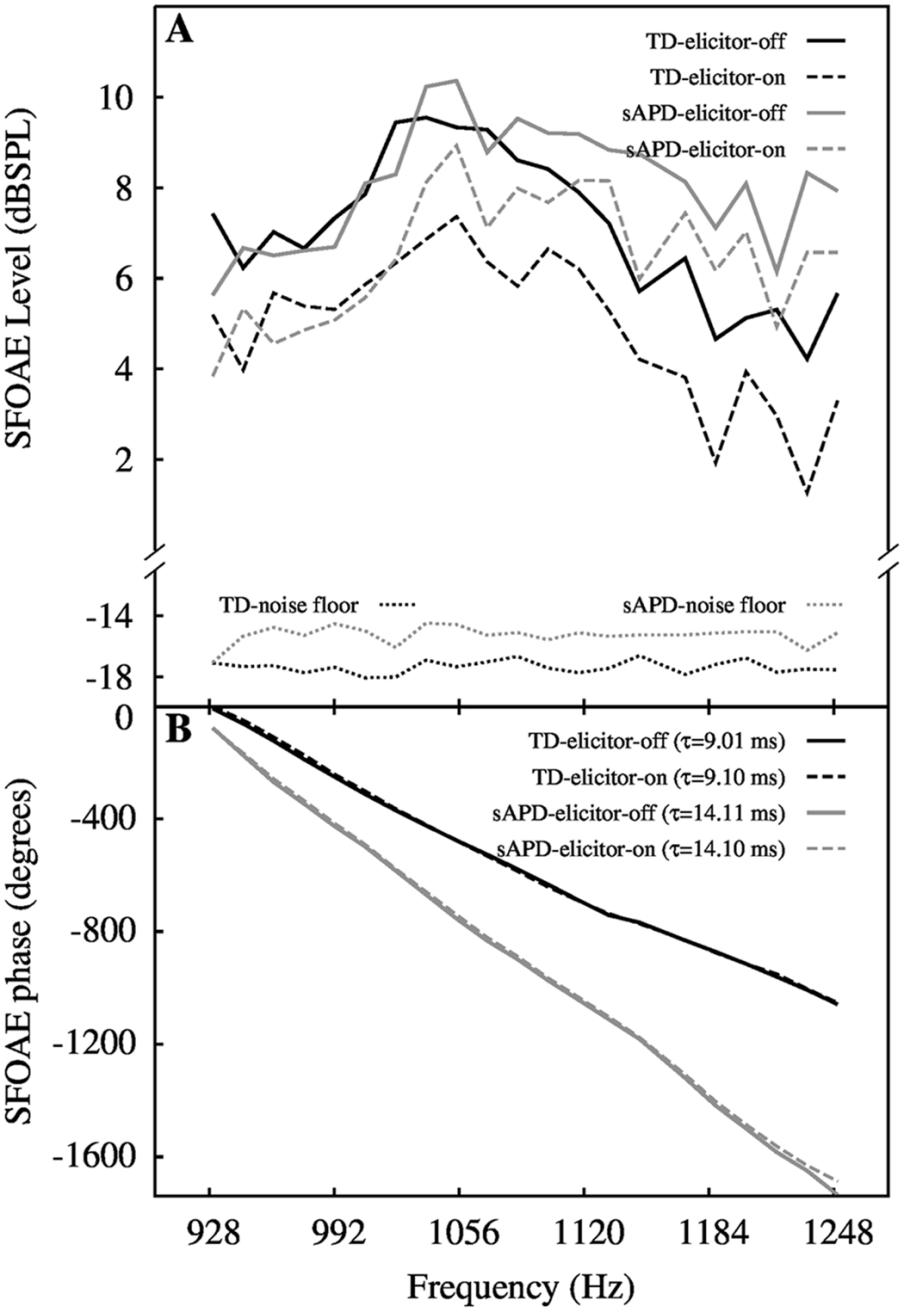
Mean SFOAE spectrum and example phase data. Panel-A shows mean (averaged across participants) SFOAE level as a function of frequency. Groups are indicated by colors (black for TD and grey for sAPD). In panel-B, SFOAE phase slopes for two children, one from TD and one from sAPD group, for elicitor-off and elicitor-on conditions are shown. The two phase gradient examples illustrate differences in the obtained *τ*. In both plots, solid lines indicate elicitor-off, and dashed lines indicate elicitor-on condition.

Mean SFOAE level for MOC elicitor-on and elicitor-off conditions across groups are plotted in Fig 3A. Examples of phase slopes, steep and shallow, one from each group, are presented in Fig 3B. Visual examination of Fig 3A shows that SFOAE level is reduced in both groups with elicitor-on. To examine if the magnitude of inhibition is different between the two groups, statistical analyses were carried out. One-way repeated measures analysis of variance (RM-ANOVA) with ‘elicitor condition’ (elicitor-on/off) as the independent variable and ‘measure’ (SFOAE level or *τ*) as the dependent variables were performed with group (TD and sAPD) as the grouping variable. Results show a significant interaction between elicitor condition and group for both SFOAE level (*F*[1, 43] = 6.76, *p* = 0.013, *η* ^2^_*Partial*_ = 0.14), and *τ* (*F*[1, 43] = 5.05, *p* = 0.030, *η* ^2^_*Partial*_ = 0.11).

**Fig 4:**
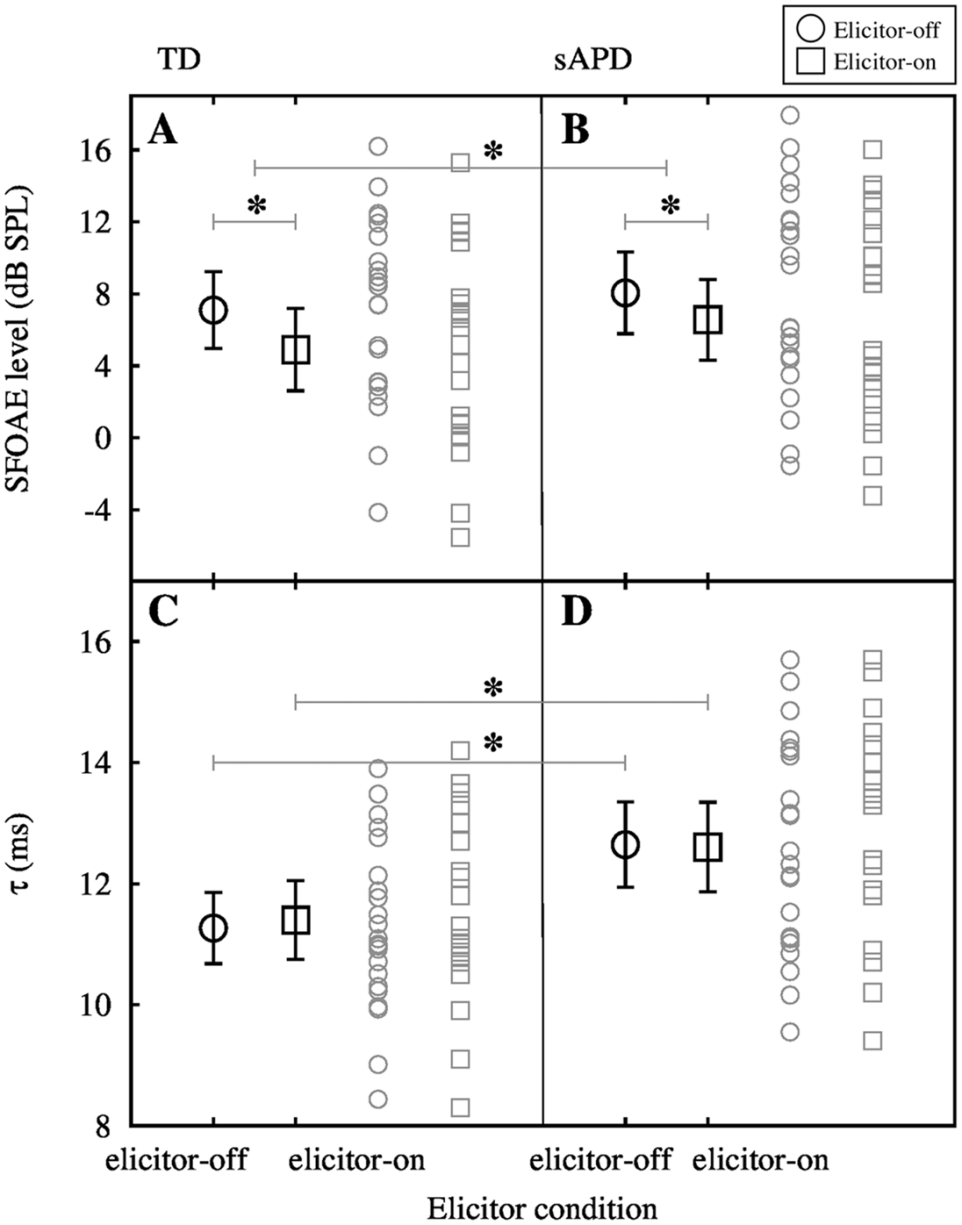
Mean MOC inhibition of SFOAE level and *τ*. Panels A and B show mean SFOAE level (averaged across participants and frequencies) for TD and sAPD groups, respectively. Error bars around the mean are 95% confidence intervals. Matching grey symbols are raw data. Panels C and D show mean *τ* for both TD and sAPD groups respectively. In both panels open circles represent elicitor-off condition and open boxes represent elicitor-on condition. Significant mean differences are marked with asterisks.

Post-hoc analyses with correction for multiple comparisons using the false discovery rate (FDR [46]) indicate significant inhibition of SFOAE level by elicitor in both TD (MD = 2.19 dB (21.71% change), CI_95%_ = ±0.51 dB, *t*[21] = 9.01, *p*<0.001), and sAPD groups (MD = 1.49 dB (15% change), CI_95%_ = ±0.40 dB, *t*[22] = 11.83, p<0.001). However, as illustrated in the top panels of Fig 4A and 4B, SFOAE level change was significantly higher in the TD group compared to the sAPD group (MD = 0.70 dB (6.13%), CI_95%_ = ±0.55 dB, *t*[43] = 2.60, *p* = 0.013). This may suggest weaker MOC functioning in the sAPD group.

### SFOAE delay with- and without- MOC inhibition

The bottom panels of Fig 4C and 4D illustrate significantly longer *τ* in the sAPD group as compared to the TD group (MD = −1.38 ms, CI_95%_ = ±0.95 ms; *t*[43] = −2.92, *p* = 0.006), possibly suggesting sharper tuning or difference in average filter shape (from TD group) in the sAPD group. Mean *τ* was 11.27 ms in the TD group and 12.64 ms in the sAPD group. *τ* in elicitor-on condition was also significantly different between groups, i.e., longer in the sAPD group (MD = −1.21 ms, CI_95%_ = ±1.02 ms, *t*[43] = −2.40, *p* = 0.021). Change in *τ* was observed in both directions, i.e., both increase, and decrease in delay from elicitor-off condition across participants.

Mean *τ* of 11.27 ms (in quiet) and 11.40 ms (with MOC activation) equate to filter bandwidths of 78.71 and 78.16 Hz, in elicitor-off and elicitor-on conditions, respectively in the TD group. In the sAPD group, mean *τ* of 12.64 ms (in quiet) and 12.60 ms (with MOC activation) equate to bandwidths of 70.37 and 70.74 Hz in elicitor-off and elicitor-on conditions. Group delay was converted to filter bandwidths using the method of Shera et al. [15].

**Fig 5:**
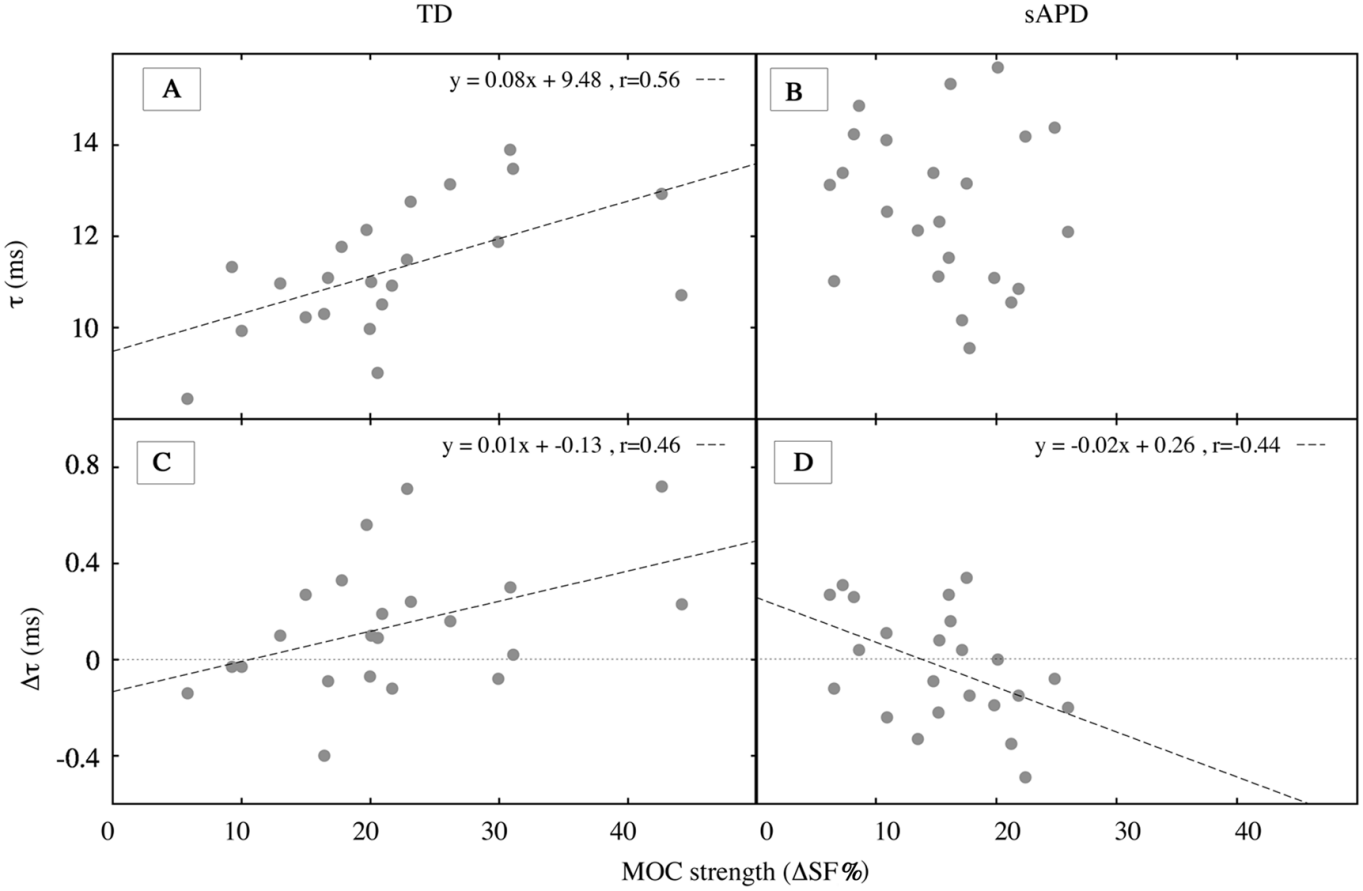
Correlation plots. Panels A and B show correlation between ΔSF and *τ* for TD and sAPD groups, respectively. Note that the correlation was significant only in the TD group. Panels C and D show correlation between ΔSF and Δ*τ* for TD and sAPD groups, respectively. In panels A, C, and D, dashed diagonal lines represent the relationship between variables in *x*- and *y*- axes and their corresponding equations are shown on the top right corner in each panel. In panels C and D, a dotted horizontal line at 0 represents no change in *τ* due to MOC inhibition. Data points above the line represent increase in SFOAE delay, and data points below the line represent decrease in SFOAE delay due to MOC activation. Notice that most children in TD group are above the 0 change line and the opposite is true in the sAPD group.

To investigate relationships between cochlear tuning and MOC functioning, a percent change measure of MOC strength (ΔSF) was subjected to correlation analysis with tuning measures (see Fig 5). ΔSF is the percent change in SFOAE level from elicitor-off condition to elicitor-on condition (re: elicitor-off) at each frequency in a linear scale (Pascal).

As illustrated in Fig 5A and 5B, correlation between ΔSF and *τ* (in quiet) was significant in the TD group (Pearson *r*[20] = 0.56, *p* = 0.006) but not in the sAPD group (Pearson *r*[21] = −0.14, *p* = 0.52). However, with elicitor-on, correlations between ΔSF and Δ*τ* were significant for both TD (Pearson *r*[20] = 0.46, *p* = 0.031), and sAPD groups (Pearson *r*[21] = −0.44, *p* = 0.035), albeit in opposite directions (Fig 5C and 5D).

Additionally, to investigate if audiometric hearing thresholds have any bearing on the SFOAE delay measures, children’s puretone average and *τ* were subjected to correlation separately for the two groups despite no significant difference between groups in their hearing thresholds. Results indicated no correlation between the two measures for both TD (Pearson *r*[16] = 0.105, *p* = 0.67), and sAPD (Pearson *r*[21] = −0.109, *p* = 0.62) suggesting that hearing thresholds probably does not have any bearing on SFOAE *τ* in both groups in the current sample.

### Noise-floor comparison

To understand reasons for higher participant rejection rate in the sAPD group, their noise-floors were compared with TD group. Mean noise-floor level in 13 of 15 children (data from two children could not be used due to excessive artifacts) that were rejected was −11.78 (SD: 5.2) dB SPL, with some as high as −3.5 dB SPL, compared to −17.21 (SD: 2.4) dB SPL in TD children who were included in the study. Pairwise comparison of noise levels between children rejected from the sAPD group and TD children revealed a significant difference (*t*[15.06] = −3.54, *p* = 0.003). Noise-floor levels were also higher in the sAPD children included in the study (Mean: −15.25, SD: 3.9 dB SPL) but were not significantly different from TD after FDR correction for multiple comparisons (MD = −1.97, CI_96%_ = ±1.96 dB SPL; *t*[36.84] = −2.05, *p* = 0.048). Note that degrees of freedom were corrected for unequal variance between groups. These differences suggest that children in the sAPD group may tend to have higher resting noise-floor compared to TD children.

## Discussion

The objective of this study was to: (1) investigate cochlear tuning, (2) reconcile MOC functioning differences between sAPD and TD children, and (3) explore the relationship between cochlear tuning and MOC functioning in children with sAPD.

### Cochlear delay and tuning

The *τ* obtained for the TD group in the current study (11.27 ms) is consistent with adult data reported in a previous study for the same frequency (1 kHz) region [40]. However, children in the sAPD group have significantly longer SFOAE delay (12.64 ms). Longer group delay may either suggest sharper cochlear tuning, or a difference in average filter shape between the two groups. In either case, the difference in delay might suggest differences in cochlear processing. The interpretation of sharper tuning may appear counterintuitive at first, because individuals with sharper cochlear tuning would typically be expected to demonstrate good listening [15, 47]. However, it should be noted that fine frequency resolution comes at the cost of temporal resolution. If a significantly longer *τ* translates to sharper tuning in the sAPD group, it could potentially affect their auditory processing in many ways. First, filter theory dictates that a sharper filter will ring longer [35]. Consequently, filter ringing in the sAPD group (2.29 ms) is 12.2% longer than the TD group (2.02 ms), where ringing duration is obtained from the bandwidth of the filter using the formula: 1/(2*π*BW) [48]. Shailer and Moore [13] reported an inverse relationship between filter bandwidth and gap detection threshold for frequencies 1 kHz and below in adult listeners. These authors suggested that ringing of the auditory filter would partially fill in the gaps in a gap detection task, limiting temporal acuity. Therefore, a 12.2% increase in ringing may lead to 12.2% poorer gap detection, and 12.2% more masking in children with sAPD. However, it is unknown if a 12.2% increase in ringing could produce noticeable effects in their speech perception. Sharper filters are a normal phenomena at lower frequencies, whereas in the sAPD group sharpness might be present even for a relatively high (1 kHz) frequency range. If longer *τ* translates to a difference in filter shapes, how this difference might impact auditory processing in the sAPD group is uncertain.

A sharper cochlear filter that rings longer may cause an increase in forward masking. In a typical speech discourse, speech sounds occur in rapid succession, and an increase in forward masking may mask speech contrasts, potentially affecting overall speech perception [49], especially for lower frequencies where filter ringing is more prominent. It may thus be speculated that longer *τ* in children with sAPD may affect their auditory temporal acuity, and in turn, speech perception and other related auditory processes. A closer look at the results of our APD test battery shows that 12 out of 23 children (sAPD group) failed the Gap Detection Test, suggesting difficulties in temporal processing in half the sample. However, there was no correlation between gap detection thresholds and *τ* (data not shown). This is not surprising given the restricted range in *τ* (9.55 to 15.7 ms) in comparison to a much larger range in gap detection thresholds (4.55 to 60.44 ms). Also, behavioral measures of temporal acuity involve coordination of several neural mechanisms and may be influenced by non-auditory factors [5].

Further, it can be hypothesized that processing aberrations at the periphery may cause a cumulative effect that could alter signal integrity at higher levels of the auditory system. For instance, given cochlear influences on auditory brainstem responses [50], atypical cochlear processing might have a bearing on some of the timing deficits reported at the brainstem level in children with listening problems (e.g., Hornickel et al. [4]). However, it should be noted that there is considerable overlap in the delay metric between the two groups. This means that not all children with listening difficulties will have longer *τ*, but perhaps a subset of APD may have listening difficulties arising due to atypical cochlear processing. Identifying children with known physiological processing differences, such as in cochlear processing, and profiling their auditory behavior may shed more light on the influence of subtle physiological differences in children with APD.

Some potential caveats to consider are middle-ear transmission differences across the age group tested in this study. However, considering there was no age effect in the delay metric obtained, this middle-ear effect can be largely ruled out. Potential stimulus level differences across individuals were accounted for with the use of individualized in-ear calibration at every frequency. Thus, even if there were ear-canal size differences or probe insertion depth differences, they were accounted for. In addition, a 10 dB SNR criterion was used in this study to avoid contamination of phase responses due to noise. Although this high SNR criterion led to rejection of many children, the remaining sample can be considered with confidence. The observed difference in delay metric observed may therefore be attributed to a difference in cochlear filter delay as implied by the coherent reflection theory [14], or differences in average filter shapes across groups.

### MOC function and cochlear processing

Significantly lower MOC strength in the sAPD group is consistent with previous studies (e.g., Muchnik et al. [19]), and is probably suggestive of reduced MOC functioning. It is enticing to interpret reduced MOC control on the cochlea in the sAPD group as responsible for their sharper cochlear tuning. This is because, reduced background MOC activity, i.e., reduced inhibitory action on the OHC function may lead to increased cochlear amplification leading to sharper tuning. However, as seen in Fig 5A, the direction of correlation in the TD group may appear to suggest otherwise: stronger MOC reflex was associated with sharper cochlear tuning. Although this interpretation appears counterintuitive, the direction of change in *τ* in the TD group seems to align well with this hypothesis. That is, at least at 1 kHz, increasing MOC strength seem to correlate with increase in Δ*τ*, suggesting a prolongation of *τ* with increasing MOC strength. Therefore, in TD group, longer *τ* in quiet may potentially be related to their stronger MOC reflex in general.

Other speculations for this relationship could be drawn from a developmental perspective. One possible reason could possibly be a pre-natal relationship between the MOC and the developing cochlea. Transient MOC innervation on the inner hair-cells has been shown to be critical for proper development of the auditory system [51]. Walsh et al. [51] reported broader tuning curves resulting from improper development of the cochlear amplifier in neonatally de-efferented cats. These investigators also suggested that the de-efferentation could lead to either over- or under- expression of a key component of the OHC amplification process. However, it is unknown if ‘reduced’, as opposed to complete loss of, MOC activity during developmental stages could lead to subtle irregularities in cochlear processing.

Another related speculation could be based on the maturity of non-linear cochlear processing. OAE-based studies report adult like intra-cochlear suppression tuning curves in full-term neonates (e.g., Abdala et al. [45]), providing support for cochlear maturity at birth. However, pre-term neonates show sharper cochlear (suppression) tuning than full-term neonates and adults for frequencies below 1.5 kHz [45]. This unusually sharp tuning has been unexplained by middle-ear transmission differences across age groups [52]. In addition, Abdala and Chatterjee [53] reported that the DPOAE input/output function in immature cochleae shows saturation at very high levels compared to adults, suggesting an extended range and ‘over-activity’ in cochlear amplification. Basilar membrane immaturity in the apical half of the cochlea [52] and immature MOC [54] have been suggested as possible causes for such atypical processing in pre-term neonates. Evidence for immature MOC activity has also been reported by Abdala et al. [41], where pre-term neonates showed persistent DPOAE level enhancements that are unrelated to DPOAE source mixing. Sharper tuning observed in the sAPD group in the current study appear similar to the sharper-than-normal tuning observed in cochleae that are thought to be immature. Reduced MOC control on cochlear amplification, and the lack of correlation between *τ* and ΔSF are congruent with the hypothesis that cochlear processing discrepancies observed in the sAPD group could stem from reduced MOC functioning with potential developmental links.

Other possible speculations that are unrelated to cochlear and MOC physiology and their maturation may also be considered. For instance, if the resting level of background MOC activity is lower in a given individual, elicitor induced MOC effect may appear to cause a larger MOC effect. While this may be unrelated to *τ*, such differences in MOC strength “headroom” may affect correlation analyses. Another consideration could be a corticofugal influence on the MOC [16] activated by the visual task (watching a silent movie). If attentional resources that influence MOC were allocated differently by children in the two groups, it may be possible that differential corticofugal effects cause differences in MOC inhibition of cochlear activity. Such potential differences could lead to different background MOC activation levels.

An intriguing result from the current study is the opposing correlation between ΔSF and Δ*τ* between groups. While MOC activation appears to prolong cochlear delay in the control group, it appears to reduce the same in the sAPD group. Considering MOC activation reduces cochlear gain, a reduction in delay can be expected due to broadening of the cochlear filter according to filter theory [18]. However, the increase in tuning found in the control group is consistent with previous studies for the 1 kHz region [37]. Using afferent fiber tuning curves in cats, Guinan and Gifford [55] showed increase in tuning upon MOC activation at low frequencies (<2 kHz). This is due to an increase in the threshold of the low frequency side of the tuning curve. At frequencies above 2 kHz, broadened tuning occurred due to an increase in threshold *at* the CF, i.e., at the tip of the tuning curve. This difference in MOC activation is suggestive of differential cochlear amplification between low and high frequency regions on the basilar membrane [56]. Similar results have also been reported for psychoacoustic tuning curves (e.g., Quaranta et al. [57]). It is possible that MOC inhibition of SFOAEs may have revealed a difference in the cochlear amplification process for the sAPD group, in addition to their sharper-than-typical tuning.

Δ*τ* in the TD group may suggest that the MOC may aid in maintaining broad tuning in quiet which promotes better temporal resolution. It can be speculated based on these findings, that in the presence of background noise, MOC inhibition of OHC activity increases cochlear delay, promoting better spectral resolution, at least at 1 kHz. Both frequency and temporal resolution are critical for optimal auditory processing. However, it is unclear how MOC inhibition for higher frequencies (>2 kHz) may produce a synchronized improvement in auditory processing upstream. Nonetheless, with these conjectures, it can be hypothesized that reduced MOC functioning in the sAPD group may potentially lead to difficulties in auditory processing, due to forward masking, and reduced signal unmasking in noise. Further studies that investigate other auditory processes in conjunction with the MOC and cochlear functioning may provide additional insights into the MOC and cochlear role in the difficulties presented by children with sAPD.

### What about rejected data?

Considering many of the children whose data were rejected belonged to the sAPD group (15 out of 18), rejected data were examined for common factors for rejection among sAPD children. All children whose data were rejected had passed the screening DPOAE test, therefore were expected to have SFOAEs at reasonably high SNRs. Indeed, many children did have such SFOAEs. However, SFOAE group delay calculation is derived from measures at multiple discrete frequencies. Therefore, if some frequencies are affected by noise or artifacts, the SFOAE group delay calculation will be unreliable. This is because the group delay calculation requires a reliable estimate of the slope of the regression line fitted to the phase data. If some frequencies are affected by large artifacts, it will render the resulting slope unreliable. This happened in 7 of 15 sAPD children who were rejected.

Comparison of noise-floor levels suggests that children with listening problems may also be quite noisy. Sources of this noise may be due to an inability to follow instruction to sit quietly, causing artifacts. Some children were unable to sit quietly even if they understood instructions. These factors point towards distractibility in sAPD children, and may have implications in an academic setting. A few children who were able to sit quietly had excessive physiological noise such as breathing and circulatory sounds. Walsh et al. [58] suggested that MOC activity mediated by attention may be involved in reducing ear-canal physiological noise. It may be possible that stronger MOC function in the TD group contributed to their lower noise-floor. Together, reduced MOC activity and co-morbidities that may be related to inattention [59] may also contribute to excessive noise in the sAPD group. Higher rejection in the sAPD group thus calls for two actions: (1) further examination of aspects such as internal physiological noise that may or may not be associated with the auditory system, (2) development of tools that are resilient to physiological noise and participant related artifacts to measure cochlear delays and MOC inhibition of OAEs.

## Conclusion

Results from the current study show atypically long SFOAE delay and reduced MOC functioning in children with listening difficulties. Increased delay may cause longer cochlear filter ringing times that can potentially affect temporal processing ability, or may point towards a general difference in auditory filter shapes. However, there were overlaps in data between the two groups, suggesting that not all children in the sAPD group will have increased cochlear delays, but perhaps a subset of sAPD may have listening difficulties arising due to longer-than-optimal cochlear delays. Correlation between SFOAE delay in quiet and MOC reflex strength in the TD group, and the lack thereof in the suspected APD group may suggest that MOC is important for normal functioning of the cochlea, even in quiet. Change in SFOAE delay in opposite directions (increase in TD, and reduction in sAPD) due to MOC activation suggests contrastive cochlear function between the two groups. Collectively, differential cochlear amplification, reduced MOC function, and significantly longer SFOAE delay may be interlinked in the findings of the sAPD group. Further studies, including with larger sample size, are required to explore auditory processes (such as speech perception) that could be influenced by such subtle differences in cochlear processing and MOC functioning in children with sAPD.

## Supporting Information

S1 Table Raw data. (XLSX)
